# Parenting children with disabilities in Sweden: a cluster-analysis of parenting stress and sufficiency of informal and formal support

**DOI:** 10.3389/fpsyg.2024.1389995

**Published:** 2024-05-31

**Authors:** Torun Täljedal, Mats Granlund, Fatumo Osman, Eva Norén Selinus, Karin Fängström

**Affiliations:** ^1^Region Västmanland—Uppsala University, Centre for Clinical Research, Västmanland Hospital Västerås, Västerås, Sweden; ^2^CHAP, Department of Public Health and Caring Sciences, Uppsala University, Uppsala, Sweden; ^3^CHILD Research Environment, Jönköping University, Jönköping, Sweden; ^4^Department of Mental Health, Norway Technical and Natural Sciences University, Trondheim, Norway; ^5^School of Health and Welfare, Dalarna University, Falun, Sweden; ^6^The Swedish School of Sport and Health Sciences, Stockholm, Sweden

**Keywords:** disabled children, parenting stress, informal support, formal support, person-oriented analysis

## Abstract

**Objective:**

To investigate patterns of parenting stress and access to sufficient informal and formal support among parents of children with disabilities. To explore whether child cognitive level, conduct problems and the need of language interpretation in contacts between parents and professionals are associated with patterns of parenting stress and support.

**Method:**

Parents (*N* = 140) of children with disabilities in Sweden completed a questionnaire about parenting stress and support. Patterns of three variables—parenting stress and access to sufficient informal and formal support—were investigated using cluster analysis. The relationship of child cognitive level, level of conduct problems and of language interpretation needs between parents and professionals to cluster membership was explored using multinomial logistic regression.

**Results:**

Five different clusters of parenting stress and support emerged. Parents in cluster 1 had lower than sample mean ratings on all three variables. Cluster 2 had elevated parenting stress, cluster 3 had elevated insufficient informal support and cluster 4 had elevated insufficient formal support. Cluster 5 had elevated ratings on all three variables. Greater child cognitive difficulties increased the likelihood of parent membership in cluster 2 (elevated stress), cluster 3 (elevated insufficient informal support), or cluster 5 (elevated ratings on all variables). Child conduct problems increased the likelihood of membership in cluster 2 (elevated stress) or cluster 5 (elevated ratings on all variables). No relationship between language interpretation needs and cluster membership was found.

**Conclusions:**

Patterns of parenting stress and sufficiency of support, and their associations with child characteristics, vary substantially. However, families of children with conduct problems experiencing elevated parenting stress in combination with insufficient informal and formal support, may be particularly vulnerable. The results of the current study highlight the clinical importance of exploring and identifying individual parenting stressors and perceived levels of support, to be able to adapt services to better suit a variety of needs, and thus promote equitable care.

## Introduction

Parents of children with disabilities often have higher levels of parenting stress than other parents (Hayes and Watson, 2013; Lee, 2013; Masefield et al., 2020), although the levels vary between families. Parenting stress can negatively impact child outcomes of early intervention (Osborne et al., 2008). It can also have negative effects on parents' mental and physical health (Olsson and Hwang, 2001; Oelofsen and Richardson, 2006; Lach et al., 2009; Miodrag and Hodapp, 2010) and on child behavior (Lecavalier et al., 2006; Neece et al., 2012; Woodman et al., 2015).

Creating and maintaining a sustainable family environment is a struggle for many families. Parents report high levels of role conflict and work- and family-role overload (Duxbury et al., 2018). Parenting stress has been conceived in various ways. The most widely used perspective, the Parent-Child-Relationship (P-C-R) theory (e.g., Abidin; Deater-Deckard, 2004) considers the domains of the parent, the child, and the relationship between them, and posits that high parenting stress leads to negative parenting behavior which in turn can increase emotional and behavioral problems in the child. The daily hassles perspective (e.g., Crnic and Greenberg) extends on the ideas of the P-C-R model (Deater-Deckard, 2004) considering the minor day-to-day events in family life that contribute to parenting stress.

Families of children with disabilities may struggle more than most to maintain a sustainable family environment because of additional adaptive demands related to their child's disability and the interaction with service systems. These struggles can be seen as stressors that can lead to parental stress (Perry, 2004). In her model of stress in families of children with disabilities Perry (2004) describes four related domains: stressors (parents' perception of child characteristics and other life events), resources (person and family system factors), supports (informal and formal), and outcomes (both negative and positive). In Perry's model, the individual and family resources and received informal and formal support mediate and/or moderate the impact of the stressors (child characteristics and other life stressors) on the negative and positive outcomes for parents. In terms of child characteristics, Perry highlights the importance of distinguishing between objective child characteristics, such as age or IQ, and parents' subjectively experienced “stressfulness” of their child's difficulties; the latter probably being more relevant to understanding parenting stress in families of children with disabilities.

In Sweden children with diagnosed congenital or early onset developmental disabilities such as autism, intellectual disability or mobility impairments are served by pediatric habilitation service teams. The services are thus delivered to a broad spectrum of children with different conditions and characteristics living in families under different circumstances. To provide services that are adapted to the needs of individual families, it is of importance to investigate the associations between perceived parental stress and important aspects of the child with disabilities and the family, such as perceived child characteristics and perceived support. In the following, children with disabilities refers to children with developmental disabilities warranting support from the Swedish habilitation services.

Challenging child behavior has in particular, been found to contribute to parenting stress in parents of children with intellectual or other disabilities (Lee, 2013; Barroso et al., 2018; Staunton et al., 2023). Challenging child behavior is related to how predictable a child is for parents and it can be seen as a child characteristic that affects how well a family can sustain family routines (Gallimore et al., 1996). Low predictability (e.g., conduct problems) in the child leads to difficulties in performing routines.

Informal support (from the family's own social network), and formal support (from professionals) have been found to be associated with lower levels of parenting stress (Guralnick et al., 2008; Halstead et al., 2018; Patton et al., 2018; Dunst, 2023) and mental and physical health problems (Lovell et al., 2012; Cantwell et al., 2014; Gouin et al., 2016), and higher levels of resilience (Peer and Hillman, 2014) and self-efficacy (Huus et al., 2017) in parents of children with disabilities. Parents with higher self-efficacy experience less support needs (Huus et al., 2017).

In their systematic review, Peer and Hillman (2014) conclude that informal and/or formal support through a stable social network is critical. They highlight the role of professionals in this, the importance of assessing these areas and proactively providing appropriate support for parents to develop and sustain resilience. Bitsika et al. (2013) found that depression and anxiety in parents of children with autism could be buffered even by low levels of resilience. In a recent meta-analysis, Dunst (2023) found both informal and formal support to be associated with greater psychological health in parents. Informal support had a greater effect than formal support. Scheibner et al. (2024) studied self-rated parenting stress among parents of children with disabilities, as well as pediatricians' estimations of the parents' stress. Almost half of the parents in the study reported a clinically relevant level of stress related to social isolation. In 85% of cases this was missed by the pediatricians.

It is possible that the importance of formal support increases when there is a lack of sufficient informal support. Families of children with disabilities are at greater risk of social isolation (Griffith and Hastings, 2014; Emerson and Brigham, 2015; Thompson-Janes et al., 2016) and may have limited access to informal support. Parents with an immigrant background may have limited access to informal support due to a reduced social network and less support from extended family and friends following migration (Khanlou et al., 2017; Xu et al., 2022). Based on Perry's model this could be seen as an “other life stressor.” In 2022, 20% of the Swedish population were born outside Sweden (Statistics Sweden, 2023). Several studies have found language and communication difficulties to constitute one of the greatest barriers of access to formal support for immigrant families of children with disabilities (Kittelsaa, 2012; Xu et al., 2022). The quality of language interpretation services varies and communication between parents and health services can be impeded despite the presence of an interpreter (Kittelsaa, 2012; Hadziabdic and Hjelm, 2019).

Parenting stress and the experiences of parents of children with disabilities is well-researched. However, studies are often limited to parents of children with a specific diagnosis and most previous quantitative research has been on a group level, limiting the understanding of within-group variance. Several earlier studies have shown that diagnosis or type of disability are relatively weak predictors of child and family functioning (Gallimore et al., 1996; Pinto et al., 2019). Thus, other variables than type of disability may provide better predictors for both child and family functioning. Parents of children with disabilities served by habilitation teams in Sweden are a heterogenous group, to study their experiences on a group level can be problematic. While informal and formal support have been found to be associated with lower levels of parenting stress and greater parental psychological health on a group level, there are likely a variety of combinations of experiences of parenting stress and perceived support among parents of children with disabilities. Finding these variations can help provide a more nuanced view of experiences within the clinical population. A person-oriented analysis explores the variation in patterns of variables among individuals, rather than how separate variables represent the most common or average variable outcomes of a population (Bergman et al., 2003).

Using cluster-analysis, this study aims to investigate patterns of parenting stress and access to sufficient informal and formal support and to explore whether child cognitive level, conduct problems and the need of language interpretation in contacts between parents and professionals are associated with these patterns. Exploring commonalities among parents with similar patterns of experiences could help provide a better understanding and identification of parents who may risk developing high levels of parenting stress. This is necessary to tailor and target support more effectively.

## Method

This study used data from the first wave (December 2020-June 2021) of the Participation and Mental Health (CHILD-PMH) longitudinal research program led by the CHILD research group at Jönköping university, Sweden. CHILD-PMH has ethical approval from the Swedish Ethical Review Authority (reference number 2019-05028 and 2020-04810). The CHILD-PMH aimed to follow the trajectories of mental health in children diagnosed with neurodevelopmental conditions and supported by habilitation teams in five regions of Sweden. Parents in the CHILD-PMH completed a questionnaire comprising demographic questions and several different rating scales. In the current study, patterns of parents' ratings of parenting stress and access to sufficient informal and formal support were investigated using cluster analysis. The association with cluster membership of the child's cognitive level, level of conduct problems, and of the need of language interpretation between parents and professionals was explored.

### Setting and participants

In spring 2020, invitations, written information, consent forms, and prepaid return envelopes were sent to parents/caregivers of all children born between 2013–2015 and 2007–2009, in contact with habilitation services in five selected healthcare regions in Sweden (*N* = 2,891). Non-response led to one reminder. All written information, invitations, reminders, and the parent questionnaire, were made available in Swedish, English, Arabic and Somali; Arabic, and Somali being two of the languages for which the habilitation services often engaged interpreters.

**Figure 1:**
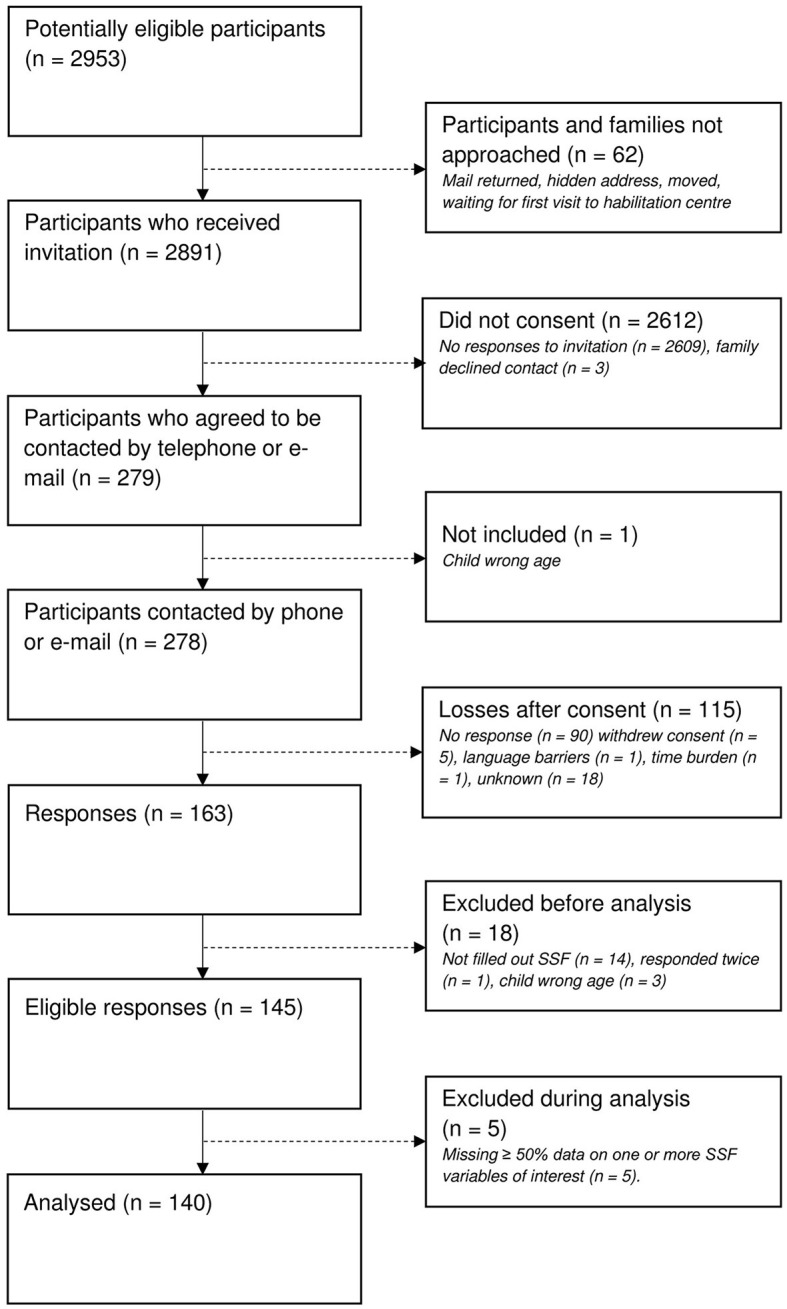
Flowchart of data collection.

COVID-19 restrictions delayed the start of data collection. In autumn 2020, all who had consented (*n* = 278) were contacted to check continued interest. Parents chose language version of the questionnaire, to be sent via e-mail link or post. Non-response after one reminder was seen as withdrawal. Figure 1 shows the data collection process. Characteristics of the children and parents are presented in Table 1. Of the 140 participants included in the final analysis, 126 (90%) used the Swedish version, eight (5.7%) the Arabic version and the remaining 6 (4.3%) the English version.

**Table 1:** Characteristics of children and responding parents.

**Child characteristic**	***n***	**(%)**
**Gender**		
Girl	47	(34)
Boy	92	(66)
Other	1	(1)
**Cognitive level**		
No difficulties	46	(33)
Mild	29	(21)
Moderate	30	(21)
Severe	21	(15)
Very severe	12	(9)
Missing	2	(1)
Age (*M, SD*)	9.64	(3.16)
**Parent characteristic**	**n**	**(%)**
**Gender**		
Male	21	(15)
Female	60	(43)
Completed by both parents together	40	(29)
Missing	19	(14)
**Education**		
< 9-year/other^*^	7	(5)
9-year elementary school	11	(8)
Upper secondary school	47	(34)
University	70	(50)
Missing	5	(4)
**Language interpretation needed in formal contacts**		
Yes, language interpretation needed	31	(22)
**Place of birth**		
Sweden	85	(61)
Europe	7	(5)
Middle East/S. Asia	30	(21)
Africa	15	(11)
S. America	2	(1)
Missing	1	(1)
**Immigrant time in Sweden**		
Number of years in Sweden *(M, SD)*	10.45	(7.76)

N = 140.

^*^5% (n = 7) did not rate their level of education but wrote an answer suggesting < 9-year elementary school, for example “illiterate,” “no education,” or “5-year school” or wrote only “SFI” (Swedish for immigrants). Their level of education was classified as “ < 9-year elementary school/other.”

### Instruments

#### Parenting stress, informal and formal support

The Strengths and Stressors in Parenting questionnaire (SSF; Ternert and Falck, 2016; Ivarsson et al., 2023) is a Swedish validated development of the Family Impact Questionnaire (Donenberg and Baker, 1993). This self-rating scale for parents, measures effects of child disability on six subscales, rated on a four-point Likert type scale [from “not at all” (0) to “very much” (3)]. Three subscales were used in this study: *feelings and attitudes about parenthood* (13 items), *impact on social life* (six items), and *contact with support system and professionals* (five items). Higher subscale scores indicate greater negative impact. In this study, the *feelings and attitudes about parenthood* subscale was used as a measure of parenting stress and the *impact on social life* and the *contact with support system and professionals* subscales were used as measures of access to sufficient informal and formal support, respectively. Internal consistency for each of the utilized subscales: *feelings and attitudes about parenthood* α = 0.84, *impact on social life* α = 0.74, and *contact with support system and professionals* α = 0.82.

#### Child cognitive level

The Ten Question Screen (TQS) is a rating scale developed for use in diverse cultures to screen for disabilities in children (Durkin et al., 1995). In the CHILD-PMH parent questionnaire, parents who answered “yes” to TQS question 10 (“Compared to other children of the same age, does your child seem to have difficulties understanding or appear mentally slow?”), were asked to answer an extra question added for the purpose of CHILD-PMH: (10a) “How would you describe the level of difficulty your child has in understanding?” with four possible answers, “mild,” “moderate,” “severe,” or “very severe.” With TQS question 10 indicating presence or absence of cognitive delay (Singhi et al., 2007), and the added question 10a indicating severity of cognitive delay, these two questions combined were used in the present study as a proxy measure of the children's cognitive level.

#### Child conduct problems

The parent-rated Strengths and Difficulties Questionnaire (SDQ; Goodman, 1997)—a globally used screening instrument for behavioral and emotional difficulties in children—has five subscales measuring various aspects of child psychological attributes and behavior on a three-point Likert type scale, from “not true” (0) to “certainly true” (2). Data was collected on all subscales but only the *conduct problems* subscale was used in the analysis for the current study, α = 0.55.

#### Language interpretation needs

In the demographic section of the CHILD-PMH parent questionnaire, parents were asked whether an interpreter was needed in contacts with habilitation services, local authorities, preschool/school etc. Their reply to this yes/no question was used as the interpretation needs variable in the analysis.

### Analysis

The authors only had access to coded data. ROPstat (Vargha et al., 2015) and IBM SPSS version 29 were used for statistical analysis. Following a residual analysis, an appropriate number of clusters was found through Ward's method of agglomerative hierarchical clustering, with a 0.7 average squared Euclidian distance from nearest neighbor. The number of clusters was chosen based on the following criteria: theoretical relevance, 5–15 clusters, explained error sum of squares percentage (EESS%) near 67%, and homogeneity coefficients < 1, as recommended by Bergman et al. (2003). K-means clustering was then performed to improve and compare solutions through case relocations. Based on the results of the hierarchical clustering, K-means clustering was carried out for four, five, six, and seven clusters. Results were compared and a final cluster solution was chosen in accordance with the specified criteria. Associations of child cognitive level, child conduct problems and the need of language interpretation between parents and professionals with cluster membership was examined through multinomial logistic regression.

## Results

### Initial analyses

Kruskal-Wallis and Mann-Whitney *U*-tests were conducted to check for differences on demographic variables. Overall, these tests revealed no significant differences based on the sociodemographic variables parent gender, parent level of education or parent born in or outside Sweden on the parents' SSF-ratings. The only exception was a significant difference between parents born outside Sweden and parents born in Sweden on the subscale Informal support (*z* = −2.22, *p* = 0.026). Residual analysis found no outliers. Prior to the multinomial logistic regression, multicollinearity was ruled out using Spearman's rank correlation (weak positive correlation between need of interpreter and child cognitive level (*r* = 0.23, *p* = 0.007), very weak positive correlation between need of interpreter and child conduct problems (*r* = 0.18, *p* = 0.037), no correlation between child cognitive level and child conduct problems).

### Patterns of parenting stress, informal, and formal support

A cluster solution with five clusters was considered to be the most appropriate solution based on the defined criteria. This solution has an Explained Error Sum of Squares percentage of 67.62%, a mean homogeneity coefficient of 0.667, point-biserial correlation of 0.343 and a silhouette coefficient of 0.645. Table 2 shows the chosen cluster solution.

**Table 2:** Profiles of SSF subscale scores, homogeneity coefficients and cluster sizes for each cluster.

**Cluster**	**Feelings about parenthood (parenting stress)**		**Impact on social life (informal support)**		**Contact with support systems and professionals (formal support)**		**HC**	**Size**
	**M**	**SD**	**M**	**SD**	**M**	**SD**		
CL1	6.03	3.84	4.24	2.30	3.13	1.97	0.65	38
CL2	16.70	4.05	6.09	2.31	6.24	1.76	0.63	29
CL3	11.97	3.59	10.57	1.74	8.04	2.50	0.66	31
CL4	7.12	3.30	4.98	1.77	9.28	2.30	0.58	24
CL5	21.56	5.02	13.50	3.15	11.63	1.50	0.91	18
Total sample	11.74	6.72	7.34	4.00	7.01	3.51		

Higher subscale scores indicate higher stress/greater negative impact on each subscale. Maximal possible values per subscale are 39, 18, and 15, respectively.

**Figure 2:**
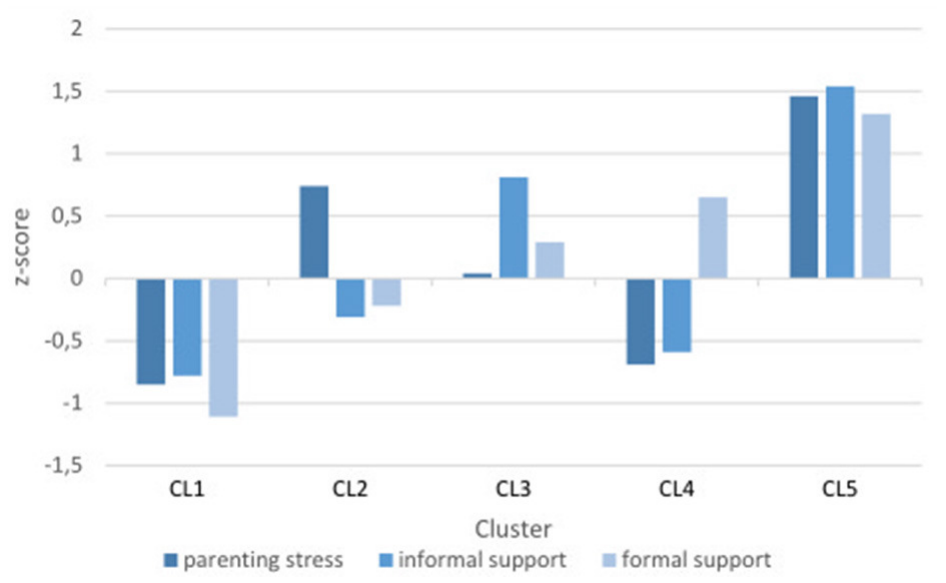
Pattern of standardized means, z-scores per cluster.

The SSF ratings indicate level of difficulty or strain on each subscale. Thus, higher ratings on the *feelings about parenthood* subscale indicate greater parenting stress, and higher ratings on the *impact on social life* and the *contact with support systems and professionals* subscales indicate greater lack of sufficient informal and formal support. Each cluster's structure can be seen in the pattern of standardized means (see Figure 2 and Table 3).

**Table 3:** Cluster characteristics.

					**Cognitive difficulties**												**SDQ conduct**		**Need of lang. interpretation**	
**Cluster**		**Pattern of standardized means (** ***z*** **-scores)**			**None**		**Mild**		**Moderate**		**Severe**		**Very severe**		**Missing**					
	***n***	**Feelings about parenthood (parenting stress)**	**Impact on social life (informal support)**	**Contact with support systems (formal support)**	***n***	**(%)**	***n***	**(%)**	***n***	**(%)**	***n***	**(%)**	***n***	**(%)**	***n***	**(%)**	**Mean**	**SD**	***n***	**(%)**
1	38	L	L	L	19	(50)	9	(23.7)	3	(7.9)	3	(7.9)	3	(7.9)	1	(2.6)	1.72	1.67	9	(23.7)
		(−0.85)	(−0.78)	(−1.11)																
2	29	H	•	•	5	(17.2)	7	(24.1)	7	(24.1)	7	(24.1)	3	(10.3)	0		3.79	1.84	7	(24.1)
		(0.74)	(−0.31)	(−0.22)																
3	31	•	H	•	7	(22.6)	7	(22.6)	9	(29)	7	(22.6)	1	(3.2)	0		1.94	2.07	3	(9.7)
		(0.04)	(0.81)	(0.29)																
4	24	L	[L]	[H]	7	(29.2)	6	(25)	8	(33.3)	2	(8.3)	0		1	(4.2)	2.04	1.55	9	(37.5)
		(−0.69)	(−0.59)	(0.65)																
5	18	H++	H++	H+	8	(44.4)	0		3	(16.7)	2	(11.1)	5	(27.8)	0		3.72	2.08	3	(16.7)
		(1.46)	(1.54)	(1.32)																

L and H indicate low and high values in relation to the sample mean, **+** indicates more extreme low or high values, **[]** indicate slight deviations from the mean and **•** indicates around the mean value for the whole sample.

### Cluster characteristics

Table 3 shows the pattern of standardized means for each cluster as well as the characteristics of each cluster in terms of the studied background variables child cognitive level, level of child conduct problems and need of language interpretation between parents and professionals.

It was not the aim of the cluster analysis to statistically compare clusters; the cluster sizes do not allow for such an analysis. Rather, the following is a description of each cluster's characteristics.

CL1 had a pattern of lower levels of parenting stress and lower levels of insufficient informal and formal support than the mean of the whole sample. This was the largest of the five clusters and the majority of the children had no or mild cognitive difficulties. This was also the cluster with the lowest ratings of child conduct problems. In 25% of cases an interpreter was needed in communication between parents and professionals.

The pattern in CL2 showed higher levels of parenting stress than the sample mean, while ratings regarding insufficient informal and formal support were around the sample mean. There was a fairly even spread of parents of children on each cognitive level. This was the cluster with the highest mean SDQ conduct rating, near the clinical cut-off level of ≥4 for children 4–17 years, according to the UK four-band cut-off solution (Youth in mind, 2016). In 25% of cases interpreters were needed in formal contacts.

CL3 had ratings in line with the sample mean regarding parenting stress, higher than the sample mean on insufficient informal support, yet in line with the sample mean on insufficient formal support. This cluster had an even spread of children on each level of cognitive difficulties. Ratings of conduct problems were the second lowest among the clusters. This was the cluster with the lowest proportion language interpretation needs.

CL4 had lower than sample mean ratings of parenting stress and slightly lower than sample mean ratings of insufficient informal support. Ratings of insufficient formal support were slightly higher than the sample mean. Children were spread evenly across the three cognitive levels: none, mild and moderate difficulties, while two children had severe difficulties. The children had SDQ conduct ratings well below clinical cut-off. This cluster had the highest proportion of interpretation needs.

Parents in CL5 rated much higher than the sample mean on each of the three SSF subscales. Almost half of the children in this cluster had no cognitive difficulties, the rest had moderate to very severe cognitive difficulties. Mean SDQ conduct ratings were near clinical cut-off. This cluster had a low proportion of interpretation needs.

### The relationship of background variables to cluster membership

In the multinomial logistic regression cluster membership was the outcome variable. Child cognitive level and child conduct problems were treated as covariates and interpretation needs as a factor. With CL1 as the reference category, child cognitive level influenced the probability of a parent being in CL2, CL3, and CL5, and level of child conduct problems influenced the probability of membership in CL2 and CL5. Greater child cognitive difficulties increased the likelihood of parent membership in CL2 (OR 1.921, 95% CI: 1.211–3.049, *p* = 0.006), CL3 (OR 1.7, 95% CI: 1.103–2.621, *p* = 0.016) or CL5 (OR 1.903, 95% CI: 1.145–3.164, *p* = 0.013) than in CL1 and greater conduct problems increased the likelihood of a parent being in CL2 (OR 1.823, 95% CI: 1.328–2.502, *p* < 0.001) or CL5 (OR 1.823, 95% CI: 1.291–2.573, *p* < 0.001) than in CL1.

## Discussion

This study aimed to investigate patterns of parenting stress and access to sufficient informal and formal support among parents of children with disabilities. It also aimed to explore whether such patterns were associated with the cognitive level and level of conduct problems of the child and language interpretation needs between parents and professionals. Five different patterns of parenting stress and support emerged. Patterns were associated with child cognitive level and child conduct problems, but not language interpretation needs.

Through a person-oriented perspective, this study adds to the knowledge base regarding the experiences of parents of children with disabilities by showing that there is a variety of patterns of parenting stress and experienced informal and formal support. This is important from a clinical perspective, to better understand the breadth of experiences among families of children with disabilities. It is important that particularly parents who experience high parenting stress and at the same time insufficient informal and formal support are identified. Parents of children with conduct problems should perhaps routinely be asked about their feelings and experiences and perceived levels of support (Lach et al., 2009; Bitsika et al., 2013). In times of limited resources and increasing numbers of families in need of support, services may become more streamlined and uniform, with less focus on the characteristics and needs of the individual family. The results of the current study highlight the importance in the clinical setting of exploring and identifying individual parenting stressors and perceived levels of support and adapting services to better suit a variety of needs to promote equitable care.

An important pattern to be aware of from a clinical perspective is the pattern of the smallest cluster (CL5). Parents in this cluster had high ratings on all three areas—parenting stress and insufficient informal and formal support. This was the smallest group, but their situation is important to understand further. Parents with high parenting stress who lack both informal and formal support may be at increased risk of negative physical and mental health (Olsson and Hwang, 2001; Oelofsen and Richardson, 2006; Lach et al., 2009; Miodrag and Hodapp, 2010), also detrimental to their child's development (Osborne et al., 2008). The probability of membership in this cluster was influenced by both child cognitive level and child conduct problems. Thus, in a clinical setting it may be especially important to ask parents of children with cognitive and conduct difficulties about their levels of stress and perceived support.

Child cognitive level, but not conduct problems, was also associated with the pattern displayed in CL3—elevated insufficiency of informal support, but parenting stress and formal support in line with the sample mean. Previous research has shown that parents of children with intellectual disability experience social isolation (Emerson and Brigham, 2015; Thompson-Janes et al., 2016), particularly if the child also has behavior problems (Griffith and Hastings, 2014). The pattern and characteristics of CL3 suggest that parents of children with cognitive difficulties also risk insufficient informal support despite low levels of child conduct problems. One possible explanation might be that a delayed cognitive development of the child affects how parents build up and sustain their social network. Many times, families tend to socialize with families with a similar social situation, e.g., being a family with young children. This allows for interaction outside the family based on the same conditions regarding child routines, e.g., bedtimes, toileting etc. Families of children with developmental delay may still need similar routines even when the child is older, while other families move on into socializing based on routines related to the needs and functioning of school aged children. When families are in different phases of the family life cycle informal networks are difficult to sustain (Hanline, 1991; DeMarle and le Roux, 2001). Most parents in CL3 had children with some level of cognitive difficulties, yet almost 23% had no such difficulties. Although a combination of child cognitive difficulties and behavior problems is a risk factor for social isolation for parents, CL3 suggests that, of course, there are also other factors that affect parents' access to informal support. What these factors may be, lies outside the scope of the current study but are important to examine further. As highlighted by Scheibner et al. (2024), professionals may focus on the disability of the child and underestimate parenting stress among parents of children whose disability is considered less severe, and other factors affecting parenting stress may be overlooked. Clinically it is thus important to discuss access to informal support with parents and perhaps together explore possible ways of expanding the parents' informal social network.

In this study greater child cognitive difficulties also increased the likelihood of cluster membership in the cluster with elevated parenting stress only (CL2). Previous research has found intellectual disability to be related to parenting stress, but again often primarily when the child also has behavior problems (Lee, 2013; Staunton et al., 2023). Indeed, child conduct problems also increased the likelihood of parent membership in this cluster. The two clusters with elevated parenting stress (CL2, CL5), were the two clusters with the highest level of child conduct problems, near clinical cut-off for the SDQ (Youth in mind, 2016). This is in line with previous research that has shown child behavior problems to increase parenting stress (Lee, 2013; Barroso et al., 2018; Staunton et al., 2023). Informal and formal support can buffer against parenting stress (Peer and Hillman, 2014). CL2 had a pattern of informal and formal support in line with the sample mean. Thus, while support can act as a buffer, there were parents with elevated parenting stress despite possibly experiencing sufficient support. This result must be interpreted with caution however, as the comparison is with the sample mean and not with the whole population of parents of children with disabilities, meaning that there is no defined base level of what constitutes sufficient informal and formal support. It could also be speculated that the level of informal and formal support experienced by parents in this cluster could be a contributing factor to the level of elevated parenting stress not being higher than it was.

The most common pattern was to have lower than sample mean ratings on all three variables (CL1). Most parents in this cluster had children without or with only mild cognitive difficulties and with low levels of conduct problems.

Language barriers have in previous research been found to affect parents' experiences of health care contacts and access to support (Kittelsaa, 2012; Xu et al., 2022). The current study did not find evidence that interpretation needs play a role in patterns of parenting stress and access to sufficient support. This could be due to the small sample size and small proportion (22%) of parents within the sample indicating language interpretation needs. Possible effects of language barriers may also be overshadowed by other issues, such as child cognitive and/or conduct difficulties. The need of interpreters does not cover all aspects of language barriers. It does not for example include parents who speak enough Swedish to manage everyday communication but may still experience difficulties in more challenging formal contacts. Families with an immigrant background may have a reduced social network following migration (Khanlou et al., 2017) and thus possibly less access to informal support. Interestingly, in the current study the two clusters with elevated ratings of insufficient informal support (CL3 and CL5) were also the clusters with the lowest proportion of interpretation needs (9.7 and 16.7%, respectively).

A strength of this study is the heterogeneity of the sample. The CHILD-PMH included parents of children with different disabilities and of different ages. Lacking proficiency in the research language is often an exclusion criterion in research. In the CHILD-PMH, measures were instead taken to enable non-Swedish speaking parents to participate. The use of person-oriented analysis is also a strength. Most previous quantitative research in the field has been variable-oriented, looking at group level variable means. A person-oriented approach allows the exploration of variation within the sample, better reflecting the breadth and variety within the clinical population. Through a person-oriented approach, patterns which are less common, but of clinical importance, can be identified.

However, the response rate was low and the representativity of the sample unknown, thus results must be interpreted with caution. The study is based on a small sample and results may not necessarily be generalizable to the larger population. Despite efforts to enable non-Swedish speaking parents to participate, there were relatively few cases where language interpretation was needed in contacts between parents and professionals. This may have affected the results of the regression.

The instruments used in the study also have some important limitations. Parents' ratings on the TQS do not give a measure of the child's cognitive level verified by professionals. It does, however, give the parents' experience of the cognitive functioning of the child. The parents' subjective experience of the child's level of functioning may be more relevant when it comes to parenting stress (Perry, 2004).

Few previous studies have used the SSF. The original idea of the SSF is to give both a measure of strengths as well as stressors, by having parents rate both positively and negatively phrased items. However, the small sample size did not allow for such sub-analyses and instead the ratings of positively phrased items were reversed for the current study. Yet, low scores on positively phrased items may not necessarily equate to high negative scores (Ivarsson et al., 2023).

Although child cognitive difficulties and conduct problems influenced cluster membership it is important to note that the odds ratios were relatively small. It may be that other factors not captured in this study in fact are more influential on patterns of parenting stress and access to sufficient support. Based on Perry's (2004) model of stress in families of children with disabilities, factors to investigate in future studies could be other life stressors such as poor housing, unemployment or financial problems as well as personal and family systems resources (Perry, 2004). This study took place during the COVID-19 pandemic which may have affected parents' ratings of informal and formal support somewhat. However, Sweden had a less restrictive response to the pandemic than many other countries. Physical distancing was recommended, and was compulsory at organized events, in restaurants, etc., but Sweden had no “lockdowns,” and schools and kindergartens remained open. The SSF asks about parents' experiences in general and not related to a specific time. So, while it is possible that the pandemic may have had some influence on the results, it was probably not great.

There are various forms of support for parents of children with disabilities in Sweden. Yet there is still, as these results show, a number of parents who have elevated parenting stress and seem to be lacking sufficient support. There are also parents who have elevated stress despite possibly not experiencing insufficiency of support, as well as parents who experience insufficient support but not elevated parenting stress. In a time when both numbers of and the diversity of families in need of support is steadily increasing while resources are limited, there is a tendency toward more group-based, one-size-fits-all interventions in formal support settings. While such interventions can be better than nothing, they often do not meet the needs of all, and there is a risk of increased health inequality (Hoddinott et al., 2010). There may be a risk in habilitation services for example, that such streamlined interventions miss the needs of families with different patterns of functioning and support needs. It is important to further explore which parents are at risk of different patterns of parenting stress and sufficiency of support and which factors contribute to this. Future research could explore the characteristics of, and conditions faced by families with different patterns to identify important contributing factors and develop services to suit different families' needs. Conduct problems in children is a risk factor for future mental health problems (Stringaris et al., 2014). Families of children with conduct problems and with parents experiencing elevated parenting stress in combination with a lack of sufficient informal and formal support may be particularly vulnerable and support for these families' needs to be secured.

## Data availability statement

The datasets presented in this article are not readily available because, data is from an ongoing project, currently stored at a protected server with code access. In 2025 when all data has been collected the data set can be “cleaned” and approved for open access and will then be stored at the university data deposit. Until then, MG is responsible for how data is handled and stored (due to rules from the Swedish Ethical Review authority and GDPR). Data cannot be provided without permission from MG or the second responsible, Lena Almqvist. In addition, approval must be sought from the Swedish Ethical Review Authority at: https://etikprovningsmyndigheten.se/en/. Requests to access the datasets should be directed to MG, mats.granlund@ju.se; Lena Almqvist, lena.almqvist@mdu.se.

## Ethics statement

The studies involving humans were approved by Swedish Ethical Review Authority. The studies were conducted in accordance with the local legislation and institutional requirements. The participants provided their written informed consent to participate in this study.

## Author contributions

TT: Conceptualization, Formal analysis, Writing – original draft, Writing – review & editing. MG: Conceptualization, Funding acquisition, Writing – review & editing. FO: Conceptualization, Writing – review & editing. EN: Conceptualization, Writing – review & editing. KF: Conceptualization, Formal analysis, Writing – review & editing.

## References

[B1] Barroso N. E. Mendez L. Graziano P. A. Bagner D. M. (2018). Parenting stress through the lens of different clinical groups: a systematic review & meta-analysis. J. Abnorm. Child Psychol. 46, 449–461. 10.1007/s10802-017-0313-6 28555335 PMC5725271

[B2] Bergman L. R. Magnusson D. El-Khouri B. (2003). Studying Individual Development in an Interindividual Context: a Person-Oriented Approach. Mahwah, NJ: L. Erlbaum Associates.

[B3] Bitsika V. Sharpley C. F. Bell R. (2013). The buffering effect of resilience upon stress, anxiety and depression in parents of a child with an autism spectrum disorder. J. Dev. Phys. Disabil. 25, 533–543. 10.1007/s10882-013-9333-537703248

[B4] Cantwell J. Muldoon O. T. Gallagher S. (2014). Social support and mastery influence the association between stress and poor physical health in parents caring for children with developmental disabilities. Res. Dev. Disabil. 35, 2215–2223. 10.1016/j.ridd.2014.05.012 24927515

[B5] Deater-Deckard K. D. (2004). Parenting Stress. New Haven, CT: Yale University Press.

[B6] DeMarle D. J. le Roux P. (2001). The life cycle and disability: experiences of discontinuity in child and family development. J. Loss Trauma 6, 29–43. 10.1080/108114401753197459

[B7] Donenberg G. Baker B. L. (1993). The impact of young children with externalizing behaviors on their families. J. Abnorm. Child Psychol. 21, 179–198. 10.1007/BF00911315 8491931

[B8] Dunst C. J. (2023). A meta-analysis of informal and formal family social support studies: relationships with parent and family psychological health and well-being. Int. J. Caring Sci. 16, 514–529.30548710

[B9] Durkin M. S. Wang W. Shrout P. E. Zaman S. S. Hasan Z. M. Desai P.. (1995). Evaluating a ten questions screen for childhood disability: reliability and internal structure in different cultures. J. Clin. Epidemiol. 48, 657–666. 10.1016/0895-4356(94)00163-K 7537327

[B10] Duxbury L. Stevenson M. Higgins C. (2018). Too much to do, too little time: role overload and stress in a multi-role environment. Int. J. Stress Manag. 25, 250–266. 10.1037/str0000062

[B11] Emerson E. Brigham P. (2015). Exposure of children with developmental delay to social determinants of poor health: cross-sectional case record review study. Child 41, 249–257. 10.1111/cch.12144 24797435

[B12] Gallimore R. Coots J. Weisner T. Garnier H. Guthrie D. (1996). Family responses to children with early developmental delays. II: accommodation intensity and activity in early and middle childhood. Am. J. Ment. Retard. 101, 215–232. 8933897

[B13] Goodman R. (1997). The Strengths and Difficulties Questionnaire: a research note. J. Child Psychol. Psychiatr. 38, 581–586. 10.1111/j.1469-7610.1997.tb01545.x 9255702

[B14] Gouin J. P. da Estrela C. Desmarais K. Barker E. T. (2016). The impact of formal and informal support on health in the context of caregiving stress. Fam. Relat. 65, 191–206. 10.1111/fare.12183

[B15] Griffith G. M. Hastings R. P. (2014). 'He's hard work, but he's worth it'. The experience of caregivers of individuals with intellectual disabilities and challenging behaviour: a meta-synthesis of qualitative research. J. Appl. Res. Intellect. Disabil. 27, 401–419. 10.1111/jar.12073 24105755

[B16] Guralnick M. J. Hammond M. A. Neville B. Connor R. T. (2008). The relationship between sources and functions of social support and dimensions of child- and parent-related stress. J. Intellect. Disabil. Res. 52, 1138–1154. 10.1111/j.1365-2788.2008.01073.x 18507703 PMC2585608

[B17] Hadziabdic E. Hjelm K. (2019). Register-based study concerning the problematic situation of using interpreting service in a region in Sweden. BMC Health Serv. Res. 19:727. 10.1186/s12913-019-4619-7 31640714 PMC6805506

[B18] Halstead E. J. Griffith G. M. Hastings R. P. (2018). Social support, coping, and positive perceptions as potential protective factors for the well-being of mothers of children with intellectual and developmental disabilities. Int. J. Dev. Disabil. 64, 288–296. 10.1080/20473869.2017.1329192 34141317 PMC8115529

[B19] Hanline M. F. (1991). Transitions and critical events in the family life cycle: implications for providing support to families of children with disabilities. Psychol. Schools 28, 53–59. 10.1002/1520-6807(199101)28:1<53::AID-PITS2310280109>3.0.CO;2-E30940178

[B20] Hayes S. A. Watson S. L. (2013). The impact of parenting stress: a meta-analysis of studies comparing the experience of parenting stress in parents of children with and without autism spectrum disorder. J. Aut. Dev. Disord. 43, 629–642. 10.1007/s10803-012-1604-y 22790429

[B21] Hoddinott P. Allan K. Avenell A. Britten J. (2010). Group interventions to improve health outcomes: a framework for their design and delivery. BMC Publ. Health 10, 800–800. 10.1186/1471-2458-10-800 21194466 PMC3022868

[B22] Huus K. Olsson L. M. Elgmark Andersson E. Granlund M. Augustine L. (2017). Perceived needs among parents of children with a mild intellectual disability in Sweden. Scand. J. Disabil. Res. 19, 307–317. 10.1080/15017419.2016.1167773

[B23] Ivarsson M. Danielsson H. Andersson A. K. Gothilander J. Granlund M. (2023). Structural validity and internal consistency of the Strengths and Stressors in Parenting (SSF) Questionnaire in parents of children with developmental disabilities. Scand. J. Psychol. 64, 486–494. 10.1111/sjop.12897 36602042

[B24] Khanlou N. Haque N. Mustafa N. Vazquez L. M. Mantini A. Weiss J. (2017). Access barriers to services by immigrant mothers of children with autism in Canada. Int. J. Mental Health Addict. 15, 239–259. 10.1007/s11469-017-9732-4 28424567 PMC5378730

[B25] Kittelsaa A. (2012). “Erfaringer fra møter mellom familier og hjelpeapparatet,” in Innvandring og funksjonshemming, ed. B. Berg (Oslo: Universitetsforlaget), 103–128.

[B26] Lach L. M. Kohen D. E. Garner R. E. Brehaut J. C. Miller A. R. Klassen A. F.. (2009). The health and psychosocial functioning of caregivers of children with neurodevelopmental disorders. Disabil. Rehabil. 31, 741–752. 10.1080/08916930802354948 19736648

[B27] Lecavalier L. Leone S. Wiltz J. (2006). The impact of behaviour problems on caregiver stress in young people with autism spectrum disorders. J. Intellect. Disabil. Res. 50, 172–183. 10.1111/j.1365-2788.2005.00732.x 16430729

[B28] Lee J. (2013). Maternal stress, well-being, and impaired sleep in mothers of children with developmental disabilities: a literature review. Res. Dev. Disabil. 34, 4255–4273. 10.1016/j.ridd.2013.09.008 24080069

[B29] Lovell B. Moss M. Wetherell M. A. (2012). With a little help from my friends: psychological, endocrine and health corollaries of social support in parental caregivers of children with autism or ADHD. Res. Dev. Disabil. 33, 682–687. 10.1016/j.ridd.2011.11.014 22186636

[B30] Masefield S. C. Prady S. L. Sheldon T. A. Small N. Jarvis S. Pickett K. E. (2020). The caregiver health effects of caring for young children with developmental disabilities: a meta-analysis. Maternal Child Health J. 24, 561–574. 10.1007/s10995-020-02896-5 32048172 PMC7170980

[B31] Miodrag N. Hodapp R. M. (2010). Chronic stress and health among parents of children with intellectual and developmental disabilities. Curr. Opin. Psychiatr. 23, 407–411. 10.1097/YCO.0b013e32833a8796 20592593

[B32] Neece C. L. Green S. A. Baker B. L. (2012). Parenting stress and child behavior problems: a transactional relationship across time. Am. J. Intellect. Dev. Disabil. 117, 48–66. 10.1352/1944-7558-117.1.48 22264112 PMC4861150

[B33] Oelofsen N. Richardson P. (2006). Sense of coherence and parenting stress in mothers and fathers of preschool children with developmental disability. J. Intellect. Dev. Disabil. 31, 1–12. 10.1080/13668250500349367 16766317

[B34] Olsson M. B. Hwang C. P. (2001). Depression in mothers and fathers of children with intellectual disability. J. Intellect. Disabil. Res. 45, 535–543. 10.1046/j.1365-2788.2001.00372.x 11737541

[B35] Osborne L. A. McHugh L. Saunders J. Reed P. (2008). Parenting stress reduces the effectiveness of early teaching interventions for autistic spectrum disorders. J. Aut. Dev. Disord. 38, 1092–1103. 10.1007/s10803-007-0497-7 18027079

[B36] Patton K. A. Ware R. McPherson L. Emerson E. Lennox N. (2018). Parent-related stress of male and female carers of adolescents with intellectual disabilities and carers of children within the general population: a cross-sectional comparison. J. Appl. Res. Intellect. Disabil. 31, 51–61. 10.1111/jar.12292 27704663

[B37] Peer J. W. Hillman S. B. (2014). Stress and resilience for parents of children with intellectual and developmental disabilities: a review of key factors and recommendations for practitioners: stress and resilience. J. Pol. Practice Intellect. Disabil. 11, 92–98. 10.1111/jppi.12072

[B38] Perry A. (2004). A model of stress in families of children with developmental disabilities: clinical and research applications. J. Dev. Disabil. 11, 1–16.19825262

[B39] Pinto A. I. Grande C. Coelho V. Castro S. Granlund M. Bjorck-Akesson E. (2019). Beyond diagnosis: the relevance of social interactions for participation in inclusive preschool settings. Dev. Neurorehabil. 22, 390–399. 10.1080/17518423.2018.1526225 30289341

[B40] Scheibner C. Scheibner M. Hornemann F. Arélin M. Hennig Y. D. Kiep H.. (2024). Parenting stress in families of children with disabilities: impact of type of disability and assessment of attending paediatricians. Child 50:e13193. 10.1111/cch.13193 37908180

[B41] Singhi P. Kumar M. Malhi P. Kumar R. (2007). Utility of the WHO ten questions screen for disability detection in a rural community-the North Indian Experience. J. Trop. Pediatr. 53, 383–387. 10.1093/tropej/fmm047 17556487

[B42] Statistics Sweden (2023). Utrikes födda i Sverige. Stockholm: Statistiska Centralbyrån. Available online at: https://www.scb.se/hitta-statistik/sverige-i-siffror/manniskorna-i-sverige/utrikes-fodda-i-sverige/ (accessed November 02, 2023).

[B43] Staunton E. Kehoe C. Sharkey L. (2023). Families under pressure: stress and quality of life in parents of children with an intellectual disability. Ir. J. Psychol. Med. 40, 192–199. 10.1017/ipm.2020.4 32106892

[B44] Stringaris A. Lewis G. Maughan B. (2014). Developmental pathways from childhood conduct problems to early adult depression: findings from the ALSPAC cohort. Br. J. Psychiatr. 205, 17–23. 10.1192/bjp.bp.113.134221 24764545 PMC4076653

[B45] Ternert M. Falck B. (2016). Föräldrastress och psykiskt välmående hos föräldrar till barn 7-12 år med funktionsnedsättning i Västerbotten (Independent thesis Advanced level (professional degree) Master's thesis). Umeå University, Umeå, Sweden.

[B46] Thompson-Janes E. Brice S. McElroy R. Abbott J. Ball J. (2016). Learning from the experts: a thematic analysis of parent's experiences of attending a therapeutic group for parents of children with learning disabilities and challenging behaviour. Br. J. Learn. Disabil. 44, 95–102. 10.1111/bld.12115

[B47] Vargha A. Torma B. Bergman L. R. (2015). ROPstat: a general statistical package useful for conducting person-oriented analyses. J. Person-Oriented Res. 1:9. 10.17505/jpor.2015.09

[B48] Woodman A. C. Mawdsley H. P. Hauser-Cram P. (2015). Parenting stress and child behavior problems within families of children with developmental disabilities: transactional relations across 15 years. Res. Dev. Disabil. 36C, 264–276. 10.1016/j.ridd.2014.10.011 25462487 PMC4425632

[B49] Xu Y. Zeng W. Wang Y. Magaña S. (2022). Barriers to service access for immigrant families of children with developmental disabilities: a scoping review. Intellect. Dev. Disabil. 60, 382–404. 10.1352/1934-9556-60.5.382 36162050

[B50] Youth in mind (2016). Scoring Instructions for SDQs for 4-17 Year Olds, Completed by Parents, Teachers or Self-report. Available online at: https://sdqinfo.org/py/sdqinfo/b3.py?language=Englishqz(UK) (accessed March 21, 2022).

